# The Age-Related *Cryptosporidium* Species Distribution in Asymptomatic Cattle from North-Western Spain

**DOI:** 10.3390/ani11020256

**Published:** 2021-01-20

**Authors:** Pablo Díaz, Esther Navarro, Susana Remesar, David García-Dios, Néstor Martínez-Calabuig, Alberto Prieto, Gonzalo López-Lorenzo, Ceferino Manuel López, Rosario Panadero, Gonzalo Fernández, Pablo Díez-Baños, Patrocinio Morrondo

**Affiliations:** Department of Animal Pathology (INVESAGA Group), Faculty of Veterinary Sciences, Universidade de Santiago de Compostela, 27002 Lugo, Spain; esther.navarro.es@gmail.com (E.N.); susana.remesar@usc.es (S.R.); dgarciadios@gmail.com (D.G.-D.); martibuig@gmail.com (N.M.-C.); alberto.prieto@usc.es (A.P.); gonzalolopezlorenzo@gmail.com (G.L.-L.); c.lopez@usc.es (C.M.L.); rosario.panadero@usc.es (R.P.); gonzalo.fernandez@usc.es (G.F.); pablo.diez@usc.es (P.D.-B.); patrocinio.morrondo@usc.es (P.M.)

**Keywords:** *Cryptosporidium*, pre-weaned calves, post-weaned calves, adult cattle, asymptomatic, north-western Spain

## Abstract

**Simple Summary:**

An age-related distribution of *Cryptosporidium* species has been reported in cattle, with the pathogenic and zoonotic *C. parvum* being predominant in suckling calves, *C. bovis* and *C. ryanae* being predominant in post-weaned calves and *C. andersoni* being predominant in adults. However, variants to this pattern have recently been reported. Unravelling the age-related species distribution pattern in cattle from a particular region will allow determining those age classes posing a higher risk to public and animal health. Thus, fecal samples from asymptomatic cattle were collected in north-western Spain. *Cryptosporidium* detection and species identification was performed by molecular methods. *Cryptosporidium* prevalence was 16.7%; it significantly decreased with age. *Cryptosporidium andersoni*, *C. bovis*, *C. occultus*, *C. parvum*, *C. ryanae* and *C. xiaoi* were identified. *Cryptosporidium parvum* was predominant in calves younger than 1 month and *C. bovis* was predominant in the rest of the age groups. The presence of *C. parvum* in all age groups implies animal and public health concerns. The predominance of *C. bovis* in cattle older than 1 month supports the idea that the age-related pattern of *Cryptosporidium* species described in cattle is not fully consistent, and thus further studies are needed to identify those factors determining the species distribution.

**Abstract:**

An age-related distribution of *Cryptosporidium* species has been reported in cattle, with *C. parvum* being predominant in suckling calves, *C. bovis* and *C. ryanae* being predominant in post-weaned calves and *C. andersoni* being predominant in adults. However, variants to this pattern have recently been reported. Thus, fecal samples (n = 594) from asymptomatic cattle were collected in north-western Spain. Animals were classified as <1 month (G1), 1–2 months (G2), 2–12 months (G3), 12–24 months (G4) and >2 years (G5). *Cryptosporidium* detection and species identification were performed by SSU rRNA PCR. Individual *Cryptosporidium* prevalence was 16.7%; it significantly decreased with age. *Cryptosporidium parvum* was predominant in G1 and *C. bovis* was predominant in the rest of the age classes; *C. bovis* and *C. ryanae* were especially prevalent in G2 and G3. *Cryptosporidium occultus* was not found in suckling calves. Finally, *C. andersoni* and *C. xiaoi* were occasionally detected in G5. The presence of *C. parvum* in all age classes implies significant animal and public health concerns. The predominance of *C. bovis* in cattle older than 1 month supports the idea that the age-related pattern of *Cryptosporidium* species described in cattle is not fully consistent, and thus further studies are still needed to identify those factors determining the species distribution.

## 1. Introduction

*Cryptosporidium* spp. are worldwide distributed apicomplexan protozoans causing gastrointestinal infections in a wide range of animal hosts, including mammals, birds, reptiles, amphibians and fish [1]. Some species mainly infect humans and spread through anthroponotic transmission (*Cryptosporidium hominis* and *Cryptosporidium viatorum*), but animals could be reservoirs of other species also related to human cryptosporidiosis outbreaks, such as *Cryptosporidium parvum*, *Cryptosporidium meleagridis*, *Cryptosporidium cuniculus*, *Cryptosporidium canis* or *Cryptosporidium felis* [1,2,3]. It has been widely demonstrated that *C. parvum* is the main zoonotic species [2,3,4], and domestic ruminants, especially cattle, are considered its most important reservoirs [5,6,7].

Application of molecular tools to the diagnosis of cryptosporidial infections in cattle has allowed the identification of more than 18 species and genotypes [8,9,10,11,12,13,14]. Due to the negative impact of cryptosporidiosis on cattle farm profitability [15], most research has focused on diarrheic outbreaks in pre-weaned calves, allowing the identification of *C. parvum* as the main species causing clinical illness [6,16,17]. However, a comprehensive analysis of available data from cattle worldwide has consistently demonstrated the presence of four major species including *C. parvum* together with the host-adapted *C. andersoni*, *C. bovis* and *C. ryanae*, mainly related to asymptomatic infections [6,18]. A number of investigations have also reported that the global distribution of these four *Cryptosporidium* species is age-related, with *C. parvum* being predominant in suckling calves, whereas *C. bovis* and *C. ryanae* are mostly found in post-weaned calves and *C. andersoni* in yearlings and adult cattle [6,12,19,20,21,22,23,24]. These results suggest that only pre-weaned calves, the major carriers of the zoonotic and pathogenic species *C. parvum*, pose a risk for both animal and public health. Nevertheless, a number of variants to this pattern have recently been reported. It has been suggested that these differences are probably due to the methodology used as well as the diverse geographic or animal management practices [25,26]. Unravelling the specific age-related species distribution pattern in cattle from a particular region will be helpful to understand the transmission dynamics of cryptosporidial infections in cattle farms as well as to determine those age classes mostly infected by zoonotic *Cryptosporidium* species and posing a higher risk to public health. In addition, identification of the major *C. parvum* carriers will allow implementing the most suitable control measures to reduce the economic impact of neonatal calf diarrhea outbreaks in cattle farms.

In Spain, the only investigations analyzing a possible age-related distribution of *Cryptosporidium* species in cattle were carried out in dairy cattle from Galicia (north-western Spain), showing an unusual pattern, since *C. parvum* was the most prevalent species in all age classes, with the presence of *C. andersoni* in heifers and adults; surprisingly, neither *C. bovis* nor *C. ryanae* were identified [27,28,29]. In order to confirm the age distribution of *Cryptosporidium* species and to provide updated and more robust data from cattle in that region, fecal samples from both pre-weaned and post-weaned calves, yearlings and adults from beef and dairy farms from the same study area (north-western Spain) were molecularly analyzed.

## 2. Materials and Methods

### 2.1. Ethics Approval Statement

All fecal samples used in this study were collected from cattle by veterinary surgeons with the permission of farm owners. All experimental procedures fully complied with ethics regulations in Spain (Royal Decree 53/2013, on the protection of animals against cruelty; www.boe.es/eli/es/rd/2013/02/01/53).

### 2.2. Study Area and Characteristics of Farms

All farms included in this study were located in Galicia (43°47′–41°49′ N; 6°42′–9°18′ W), in the northwest of Spain. This region is a very important livestock breeding area with both dairy and beef cattle raising being the most important livestock production; in fact, Galicia is the Spanish region with the highest census of dairy cattle as well as one of the most important beef cattle breeding areas of the country [30]. Nevertheless, the herd size is low, with a mean number of nineteen animals per farm [31]. Most dairy and beef cattle in Galicia are reared in small traditional family farms where animals go to pastures daily, although a number of dairy cattle farms have been modernized in recent years through the acquisition of farming machinery and better intensive production facilities, leading to an increase in herd size and productivity [31].

### 2.3. Sampling of Animals

A total of 594 fecal samples were collected in 86 and 60 dairy and beef Galician cattle farms, respectively, between 2016 and 2018. All farms were registered in cattle health defense associations (ADSG) and implemented a sanitary program. Selection of farms was performed by the veterinary surgeons responsible for the ADSGs, including only farms where no diarrheic outbreaks had been registered in suckling calves during the 12 months before sampling and no preventive treatments against *Cryptosporidium* spp. (halofuginone or paromomycin) had been administered. Individual fecal samples were taken directly from the rectum by the ADSG veterinary surgeons. No animals showed gastrointestinal clinical signs when sampled. Fecal consistency was scored according to Ireland-Perry and Stallings [32]; all samples were firm to soft (scores 3–4) and thus considered non-diarrheic. Animal age was obtained from official documentation. The age of sampled animals ranged from 2 days to 16.8 years old. Five groups were formed considering the age of the animals: suckling calves younger than 1 month (G1; *n* = 108); pre-weaned calves aged 1–2 months (G2; *n* = 62); post-weaned calves aged 2–12 months (G3; *n* = 96); yearlings aged 12–24 months (G4; *n* = 116); and adults older than 2 years (G5; *n* = 212). Sample size was calculated for estimating the true population proportion in each group with a confidence interval of 95% (α error) and 90% precision (β error), according to previous reports in cattle from the same study area [29,33]. Fecal samples were kept at 4 °C and processed within 48 h after collection.

### 2.4. Molecular Methods

*Cryptosporidium* oocysts were firstly concentrated from 2 g of feces using a diphasic sedimentation technique as previously described [34]. Then, 200 mg of the sediment was subjected to three cycles of freeze–thaw (−196 °C for 1 min and 100 °C for 7 min) and later *Cryptosporidium* DNA was extracted using a commercial kit (Realpure Spin Food-Stool Kit; Real, Valencia, Spain) following the manufacturers’ instructions. Purified DNA was stored at −20 °C until used.

In order to detect the presence of *Cryptosporidium* DNA, a nested PCR targeting the small subunit ribosomal RNA (SSU rRNA) gene was performed, using previously described primers and protocols [35]. PCR products were separated after electrophoresis in 1% agarose gels and visualized in a Fluor-S Multimager (Bio-Rad, Hercules, CA, USA) after staining with RedSafe (INTRON biotecnology, Gyeonggi, Korea). Positive samples were directly sequenced in both directions in the Sequencing and Fragment Analysis Unit of the University of Santiago de Compostela using an ABI 3730xl sequencer (Applied Biosystems, Foster City, CA, USA) and sequences were aligned and edited with Chromas Pro (Technelysium, Brisbane, Australia). Finally, consensus sequences were searched against the GenBank database using the Basic Local Alignment Search Tool (BLAST; https://blast.ncbi.nlm.nih.gov/Blast.cgi) to identify the species/genotypes present. When SSU rRNA sequences were not informative enough for reliable species identification, a further sequence analysis at the actin gene was performed [36].

Those isolates identified as *C. parvum* were also characterized at the *gp60* gene using a nested PCR previously described [37]. Positive samples were sequenced as previously described, and subtypes were named as previously proposed [38].

### 2.5. Statistical Analysis

A one-sided decreasing Cochran–Armitage test was used to check a trend in the *Cryptosporidium* prevalence. A *p*-value ≤ 0.05 was required for significance. All statistical procedures were conducted with R statistical language (R version 4.0.3, R Statistical Computing, Vienna, Austria), using the CochranArmitageTest() function from the DeskTools package.

## 3. Results

*Cryptosporidium* DNA was detected in 99 out of 594 samples (16.7%), and in 44.5% of farms, at least one positive animal was found. Sequence analysis allowed the identification of six *Cryptosporidium* species, with *C. parvum* (42/99) and *C. bovis* (36/99) being the most frequent, followed by *C. ryanae* (10/99) and *C. occultus* (7/99). *Cryptosporidium andersoni* (2/99) and *C. xiaoi* (1/99) were only occasionally found. One sample showed fainted bands and could not be correctly sequenced.

All the obtained partial SSU rRNA sequences were identical to the others deposited in the GenBank database. All *C. andersoni* sequences were 100% homologous to KT922228, and those identified as *C. parvum* matched with GQ983351, KU679364, KC886318 and MF589922. Most *C. ryanae* sequences were identical to KT922234, whereas a single *C. ryanae* sequence was identical to KY711520. Similarly, most *C. bovis* sequences coincided with MF074602, while two isolates were identical to KC618608 and EU827365, respectively. All *C. occultus* SSU rRNA sequences matched with MK982467, but they were also very similar (2 bp discrepancies) to the *C. suis* sequence JQ936502. For this reason, *C. occultus* identification was confirmed at the actin gene; our sequences were 100% homologous to MG699170, showing more than 20 bp discrepancies with *C. suis* sequences (AB852579). Finally, DNA sequencing of the SSU rRNA gene of the *C. xiaoi* isolate yielded a partial sequence with 100% similarity to *C. xiaoi* (KY055405) and *C. bovis* (EU827365) sequences; nevertheless, it matched with the *C. xiaoi* GU553017 sequence at the actin gene, showing an inter-isolate variance of 18 bp when compared with *C. bovis* AY741307.

**Figure 1:**
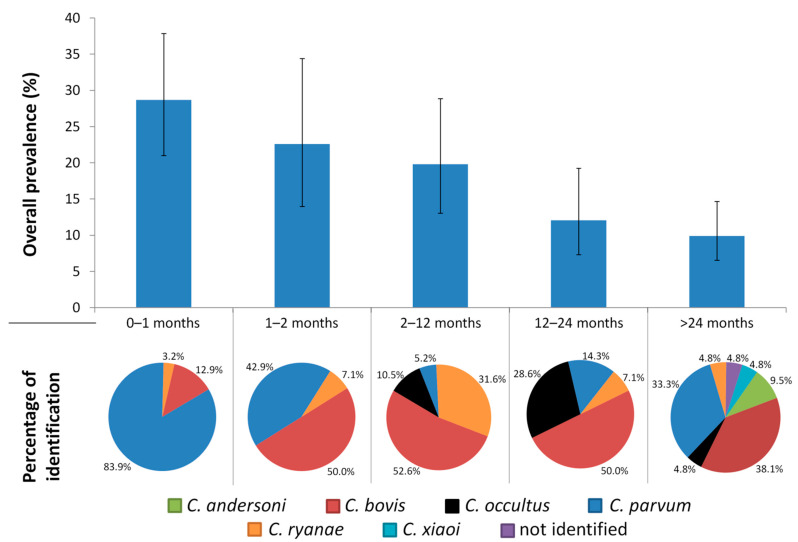
Prevalence of *Cryptosporidium* spp. and percentage of identification of *Cryptosporidium* species detected in healthy cattle from Galicia when the age of the animals was considered.

The percentage of *Cryptosporidium*-positive animals significantly decreased (Z = 4.663; *p* < 0.001) with age and ranged from 28.7% in calves younger than one month to 9.9% in adult cattle (Figure 1). In contrast, the variability of *Cryptosporidium* spp. increased with age and ranged from three species found in calves younger than two months to six species identified in adult cattle (Figure 1). Only three species (*C. bovis*, *C. parvum* and *C. ryanae*) were detected in all age classes. *Cryptosporidium parvum* was the predominant species in the youngest calves (G1), although *C. bovis* was the most common in the rest of the age classes.

The prevalence of the six *Cryptosporidium* species found showed different patterns when considering the age classes (Figure 1). Suckling calves were the most commonly infected by *C. parvum*, especially G1 calves younger than one month. *Cryptosporidium bovis* was especially prevalent in calves aged 1–24 months (G2, G3, G4) and *C. ryanae* predominated in calves aged 2–12 months (G3). *Cryptosporidium occultus* was found in cattle older than 2 months and two species (*C. andersoni* and *C. xiaoi*) were detected sporadically and only in adult cattle.

Subtyping at the *gp60* gene was successful in 30 (71.4%) *C. parvum* isolates; seven samples generated unreadable electropherograms, while five samples resulted negative (Table 1). Two subtypes belonging to the allele family IIa were detected: IIaA15G2R1 was the most common, whereas IIaA16G3R1 was only sporadically found (Table 1). All IIaA15G2R1 and IIaA16G3R1 sequences were identical to reference sequences JF727755 and DQ192506, respectively. The subtype IIaA15G2R1 was found in all age classes except for G3 calves aged 2–12 months (Table 1). In contrast, IIaA16G3R1 was only detected in calves younger than 12 months.

## 4. Discussion

Our results reveal that *Cryptosporidium* spp. is a prevalent parasite in asymptomatic cattle from north-western Spain. The percentage of infected animals found in the present study (16.7%) is consistent with previous studies in Spain and other countries worldwide reporting infection rates between 10% and 19% in healthy cattle [23,26,29,33,39,40,41,42,43], although infection rates up to 45% have also been found [10,44,45,46,47]. These variations could be a consequence of geographical differences in the prevalence of *Cryptosporidium* infections, although other factors related to the sampling methodology, age of animals, herd size and management, hygiene of facilities or season of sampling may also be involved [48].

The prevalence of *Cryptosporidium* infection was significantly highest in calves under 1 month of age and decreased progressively with age, which agrees with most studies indicating that this protozoan is most common in suckling calves [12,21,23,29,33,40,46,48,49,50]. It has been suggested that newborn calves are more susceptible to cryptosporidial infections due to their immature immune system [20], and the reduction in prevalence rates with age could be due to the development of a partial protective immune response after multiple infections with the protozoan [50]. In fact, a number of studies performed in several European countries, the USA, Brazil, China and Japan have shown that *Cryptosporidium* infection prevalence in healthy cattle older than 2 years is usually lower than 10% [20,23,28,29,33,39,40,46,51,52,53,54].

The six *Cryptosporidium* species identified in the present study have been previously reported in cattle. Four species (*C. parvum, C. bovis, C. ryanae* and *C. andersoni*) are considered the most common in cattle worldwide [3,6,18], while *C. occultus* and *C. xiaoi* have been sporadically detected [11,55]. Previous studies in China, the UK and the USA have shown age-related variations in the distribution of different *Cryptosporidium* spp. in both dairy and beef cattle, with *C. parvum* being responsible for most infections in pre-weaned calves under 2 months of age, whereas *C. bovis* and *C. ryanae* are found predominantly in post-weaned calves and *C. andersoni* is much more prevalent in yearlings and adult cattle [6,18,22,23,24]. However, other studies have found that cattle of all ages are susceptible to different *Cryptosporidium* species, suggesting that this may not be an age-related pattern. Thus, *C. bovis* has been reported as the most common species found in pre- and post-weaned calves in some cattle farms from China, India, Japan, Sri-Lanka, Sweden, the USA and Vietnam, whereas *C. parvum* is absent or present in low proportion [22,25,44,51,56,57]. Marked differences have been observed in cattle from some regions of China and Brazil where *C. andersoni* is present in all age classes and clearly predominant in animals older than 3 months of age [10,14,39,49,58,59].

The current study reveals differences in the age-related pattern abovementioned. *C. parvum* was the most prevalent species and predominated in calves younger than 1 month, but *C. bovis* was the most common in the remaining age classes, including calves aged 1–2 months and adult cattle. The role of *C. parvum* in the etiology of neonatal calf diarrhea is well recognized [6] and a previous study in this geographical area has shown a high infection rate of this species in diarrheic pre-weaned calves [16]. Small traditional family farms predominate in this geographical area, where the low density of animals may favor a progressive infection with a low number of oocysts if adequate hygienic and management measures are implemented, explaining the noticeable number of positive herds with no records of neonatal diarrhea in the last year [60]. Nevertheless, this study indicates that *C. parvum* is widely disseminated in asymptomatic cattle since it was found in all age classes and was the second most common species in adult cattle, which could play a role in farms as reservoirs of *C. parvum* for highly susceptible newborn calves. These findings also support the role of cattle of any age as a significant source of this zoonotic *Cryptosporidium* species for humans.

*Cryptosporidium bovis* is the second most commonly reported species in cattle worldwide, followed by *C. ryanae* [61]. Unlike *C. parvum*, these species have not been associated with diarrhea in cattle [62] and are mainly found in post-weaned calves and heifers [12,19,20,23,25,40,42,44,51,54,63,64]. Nevertheless, a few studies from China, Canada, India and Sweden have shown a dominance of *C. bovis* in pre-weaned calves, revealing that the age-related occurrence is not well defined and varies according to different factors, such as the geographic location or the herd management system [25,54,65,66,67]. In a beef herd monitored over three consecutive years, different species patterns were observed, with a predominance of *C. bovis* in the first year and a pre-dominance of *C. parvum* in the last year [68]. In this study, *C. bovis* was the second most prevalent species and was found in all age classes but predominated over *C. parvum* in calves aged 1–12 months, yearlings and adults. *Cryptosporidium ryanae* was sporadically detected in all age classes, which is consistent with the lower prevalence reported for this species worldwide, although most infected animals were those within the age range of 2–12 months [19,23,40,42,44,51,63]. It is worth mentioning that these two host-adapted species had not been reported in previous studies in cattle of different ages in Galicia [27,28,29]. Earlier studies have found that the infection dynamics of *C. bovis/C. ryanae* may be affected by the presence of *C. parvum*. In regions where *C. parvum* is endemic, such as the study area [16,33,34,35], it has been suggested that the high infection rate and shedding intensity of *C. parvum* in suckling calves younger than 1 month may conceal the concurrent infection of these animals by *C. bovis* or *C. ryanae* [25]. In dairy farms known to be free from *C. parvum*, the excretion of these species has been reported to start in the first three weeks, and the cumulative incidence of *C. bovis* reached 100% when the calves were five weeks old [67,69]. This dynamic has also been found in cattle reared in traditional husbandry systems in developing countries, where *C. bovis* and *C. ryanae* appear early in calves in the absence of *C. parvum* [70,71].

*Cryptosporidium andersoni* was identified in only two adult cows in this study, which agrees with previous reports in north-western Spain reporting a low prevalence for this species [27,28,29]. Some studies have concluded that *C. andersoni* only infects adult cattle, being considered the predominant *Cryptosporidium* spp. in this age class [4,19,20,23,53]. However, other studies have found a broader age range for *C. andersoni* [10,39,41,44,45,49], which has even been reported as the most prevalent species in suckling and post-weaned calves, heifers and adult cattle in some countries, especially Brazil and China [26,39,52,54]. It is significant to note the finding in this study of seven animals infected by *C. occultus*. This species was previously reported as *C. suis*-like in humans, cattle, water buffalo and domestic yaks [55] and has recently been identified in humans and rats in China [72,73,74]. Nevertheless, rats seem to be the primary host for this *Cryptosporidium* spp. and it has not been found infectious for calves under experimental conditions [55]. In farms with poor rodent control, it has been suggested that *C. occultus* may be only an intestinal transient in cattle as a result of the ingestion of food contaminated with feces of infected rodents [6]. In this study, most *C. occultus*-positive cattle (6/7) were post-weaned calves, heifers and adults from a single farm and fed with solid food placed on the ground of the farm, which increases the risk to be contaminated with rodent feces. In contrast, this species was not seen in suckling calves, which are mainly fed using milk feeders, and, consequently, all utensils used for that are more difficult to be contaminated with rodent feces.

*Cryptosporidium xiaoi* is considered one of the most common *Cryptosporidium* spp. in small ruminants, being responsible for over 90% of cryptosporidial infections in sheep and goats together with *C. ubiquitum* [61]. It has been suggested that both species are more likely to be found in healthy lambs, whereas *C. parvum* predominates in clinically ill lambs [63]. Reports of infections by *C. xiaoi* in cattle are limited worldwide, with sporadic infections being reported in Jordan and China [49,75]. In Ireland, some infections in cattle were identified as *C. bovis/C. xiaoi* [11], given the sequence similarity of both species at the SSU rRNA gene that makes accurate differentiation between both species in some cases difficult [76]. The finding of *C. xiaoi* may be related to cross-infections due to the traditional livestock farming system used in a number of Galician livestock farms, where cattle, sheep and goats share pastures and facilities [34,77]. Earlier studies in this geographical area have associated *C. xiaoi* with neonatal diarrheic outbreaks in goat farms in this geographical area [77,78], and it was also identified in asymptomatic sheep and goats [34].

Our results also have public health implications since some *Cryptosporidium* species detected in asymptomatic cattle have been previously identified in human samples. In this respect, the most frequent species identified, *C. parvum*, is considered the most important zoonotic species and, together with *C. hominis*, responsible for most of the human cryptosporidiosis outbreaks worldwide [2,3,18]. In addition, asymptomatic cattle from Galicia shed *C. parvum* oocysts belonging to subtypes IIaA15G2R1 and IIaA16G3R1, previously identified as the major *C. parvum* subtypes found in cryptosporidiosis outbreaks in calves from Spain [16,17]. Subtype IIaA15G2R1, considered one of the major *C. parvum* subtypes causing human cryptosporidiosis outbreaks worldwide [18], was predominant, whereas the latter seems to play a minor zoonotic role since it has been identified in a limited number of cattle and humans [16]. *Cryptosporidium andersoni* has been sporadically detected in humans and thus considered a species of minor public health significance [4,6]; nevertheless, it has been identified as the most common *Cryptosporidium* species in human patients from some regions of China [2]. In addition, *C. bovis* and *C. occultus* were also found in a limited number of human samples [2,55], although their pathogenicity for humans has not been described yet. Considering all these results, asymptomatic cattle, especially suckling calves under 1 month, could act as reservoirs and disseminators of some zoonotic *Cryptosporidium* species; thus, these animals could have a significant impact on public health. In fact, a previous investigation performed in the study area showed a strong predomination of *C. hominis* (65.4%) and IIa subtypes of *C. parvum* (30.0%) in human samples [79]. In addition, the identification of *C. parvum* and *C. andersoni* oocysts in river water samples from Galicia [80], as well as the finding of *C. parvum* in mussels not subjected to depuration from Galician estuaries [81], strengthens the importance of cattle as a potential source of environmental contamination with zoonotic *Cryptosporidium* oocysts. Thus, people in close contact with cattle should implement those suitable hygienic measures to avoid getting infected; cattle farmers should also apply measures to avoid oocyst dissemination in the environment.

## 5. Conclusions

Our results reveal that *Cryptosporidium* spp. are prevalent parasites in healthy cattle from north-western Spain, with the pathogenic and zoonotic species *C. parvum* being the most frequently identified species. Its finding in all age classes implies significant animal and public health concerns. Although healthy calves under 1 month are the major carriers of *C. parvum*, adult cattle may also play a role in the appearance of cryptosporidiosis outbreaks in calves and humans. The predominance of *C. bovis* in cattle older than 1 month supports the idea that the age-related pattern of *Cryptosporidium* species described in cattle is not fully consistent, and thus further studies are still needed to identify those factors determining the species distribution.

## Figures and Tables

**Table 1:** *Cryptosporidium parvum* subtypes identified in cattle from Galicia (north-western Spain) when the age of the animals was considered.

Age Class (Months)	Number of *C. parvum* Positives	IIaA15G2R1 *n* (%)	IIaA16G3R1 *n* (%)	Not Identified *n* (%)
G-1 (0–1)	26	18 (69.2)	2 (7.7)	6 (23.1)
G-2 (1–2)	6	4 (66.7)	1 (16.7)	1 (16.7)
G-3 (2–12)	1	0 (0)	1 (100)	0 (0)
G-4 (12–24)	2	1 (50)	0 (0)	1 (50)
G-5 (>24)	7	3 (42.9)	0 (0)	4 (57.1)
Total	42	26 (61.9)	4 (9.5)	12 (28.6)

## Data Availability

The data presented in this study are contained within this article.
